# Climate change/variability and hydrological modelling studies in Zimbabwe: a review of progress and knowledge gaps

**DOI:** 10.1007/s42452-021-04512-9

**Published:** 2021-04-12

**Authors:** Auther Maviza, Fethi Ahmed

**Affiliations:** 1 Department of Environmental Science and Health, Faculty of Applied Sciences, National University of Science and Technology, Corner Cecil Avenue and Gwanda Road, Ascot, Bulawayo Zimbabwe; 2 School of Geography, Archaeology and Environmental Studies, University of Witwatersrand, 1 Jan Smuts Avenue, Braamfontein, Johannesburg, South Africa

**Keywords:** Zimbabwe, Climate change, Hydrology, Modelling, GIS, Remote sensing, 86A08, 86A05, AR14, Q24, Q25

## Abstract

This paper reviews developments in climate science and hydrological modelling studies in Zimbabwe over the past 29 years in an effort to expose knowledge gaps within this research domain. We initially give a global and regional overview and then follow a systematic thematic approach in reviewing specifically online published, peer-reviewed journal articles on climate change/variability and hydrological modelling in Zimbabwe. The state and progress towards advanced integrated climate and hydrological modelling research are assessed, tracking benchmarks in the research methodologies (tools and techniques) used therein including geographic information systems and remote sensing. We present descriptive summaries of key findings, highlighting the main study themes (categories) and general conclusions arising from these studies while examining their implications for future climate and hydrological modelling research in Zimbabwe. Challenges associated with climate and hydrological modelling research in Zimbabwe are also briefly discussed and the main knowledge gaps in terms of research scope and methodologies employed in the reviewed studies also exposed. We conclude by presenting plausible potential areas of focus in updating and advancing scientific knowledge to better understand the climate-land use-hydrology nexus in Zimbabwe. While this paper is primarily relevant for researchers, the general findings are also important for policy-makers since it exposes potential areas for policy intervention or agenda setting in as far as climate and hydrology science research is concerned so as to effectively address pertinent questions in this domain in Zimbabwe.

## Introduction

A review of global climate changes since 1700 has revealed that over the centuries, twenty climatic events covering continental-scale temperature fluctuations, hydroclimatic anomalies, stratospheric perturbations and general atmospheric composition changes have occurred, impacting millions of people in many ways [1–4]. As such, understanding and predicting these inter-annual, and multi-decadal variations and changes in climate and the resultant impacts has become a critical and active area of research globally over the decades. Several studies have been undertaken to quantify the extent of impacts and the dynamics (in space and time) of climate change on water resources [5–11], food security [12–16], ecosystems [17–20], energy, and human health [21–23]. All these studies have revealed that climate change is a significant factor to consider in holistic planning for community resilience and adaption, fostering global progress towards achieving the United Nations Sustainable Development Goals (UN SDGs), Agenda 2030 and Paris Agreement goals [24–27]. These impacts are expected to vary in different countries in various regions of the world considering the differences in climate-sensitivity of vulnerable populations with likely increases in poverty and inequities as a consequence of climate change especially in developing countries [28, 29].

In developing countries in Africa for example, where the impacts of climate system changes are predicted to be manifest in more uncertain terms [30–37], expanding knowledge in this domain has become more pertinent hence the steady developments in research therein. In Southern Africa, studies also indicate a continued high climate variability [35, 38, 39] marked by recurrent droughts and floods in the future [40–43], notwithstanding the uncertainties in these studies. The scope of these studies has been diverse, covering various focus areas such as climate modelling [44–47], hydrological impacts [48–51] and other general impact studies [52–55].

Despite all the advances made in the aforementioned studies, knowledge gaps are well acknowledged particularly considering the inherent uncertainties in the new developments in climate science/modelling and climate impact assessment techniques [56]. Tools and approaches are now available and more are being developed that allow for a better understanding and characterisation of the implications of climate change and variability to assist in better climate risk management strategy development [57, 58] in developing countries such as Zimbabwe. As such, the scientific community within and outside Zimbabwe has, over the past decades, been able to exploit various tools and techniques to generate new knowledge pertaining to the local climate dynamics and impacts to better guide decision making specifically tailored to the local needs. One key area of focus has been the implications of climate change on water resources/hydrological systems, considering that a significant part of Zimbabwe is generally semi-arid in nature.

Furthermore, considering the acknowledgement of spatio-temporal land use and land cover change (LULCC) as an important factor (with both direct and indirect implications) on hydrological systems [59–64], attempts have also been made to explore the climate-LULCC-hydrology interlinkages using coupled systems approaches in various studies globally [65–69]. All these studies indicate a wide scope of themes covered over the years as earlier mentioned and as such, it becomes important to explore and characterise these studies in a more systematic manner so as to better appreciate the advances made so far and identify the knowledge gaps therein. Very few known studies, e.g. Bhatasara [70] and Brazier [71], have attempted to extensively review climate change research in Zimbabwe albeit from a Foucauldian discourse perspective and general impacts and mitigation sense, respectively. With regards to climate-hydrological modelling studies, no known study has reviewed such; hence in this paper we attempt to expand the scope of review in these areas by presenting key research developments in climate science and hydrological modelling in Zimbabwe over the past three decades. The ultimate goal is to expose knowledge gaps, i.e. possible areas for further research in this domain in Zimbabwe.

## Methods

**Fig. 1:**
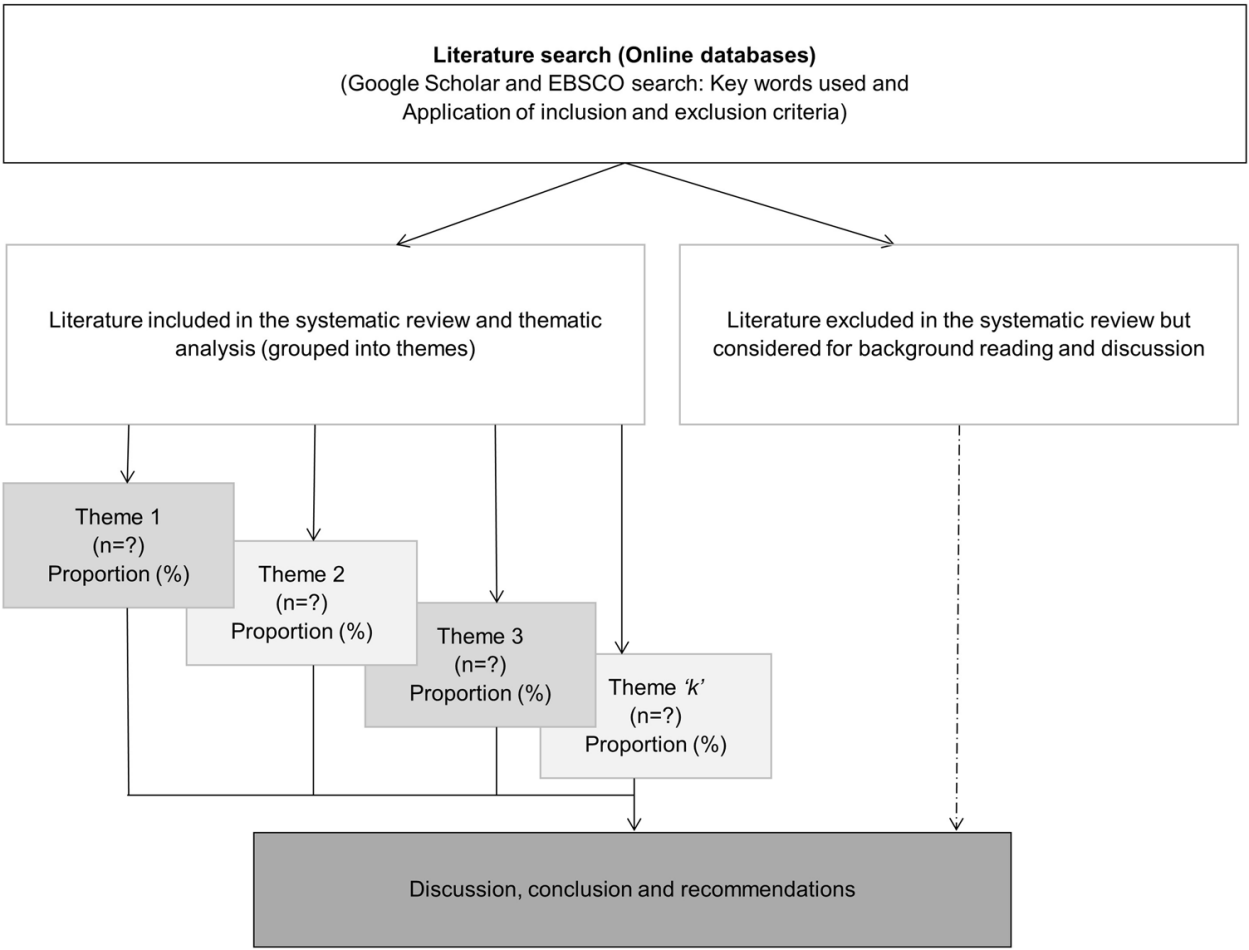
Methodology flow diagram showing the main steps of the study approach. ( Adapted from Nichols et al. [74])

**Fig. 2:**
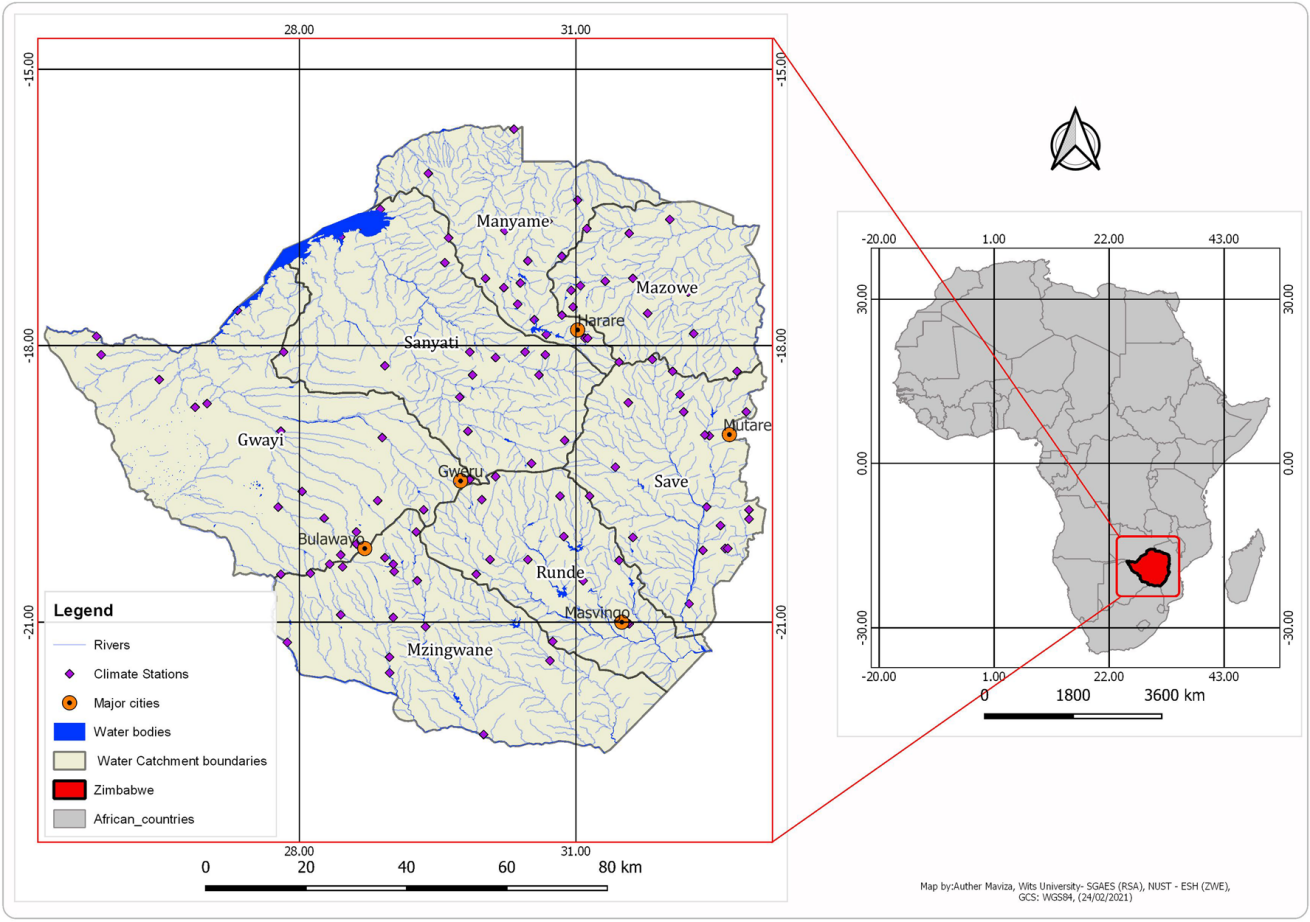
Map showing location of Zimbabwe (study area) relative to other countries in Africa

We adopted a systematic search for relevant peer-reviewed literature from a range of databases searched using Google Scholar (GS) search engine leveraging its strength of cataloguing 100 million records of academic literature and most importantly being able to competently find potentially valuable grey literature (i.e. articles published by non-commercial academic publishers) [72]. We also utilised EBSCO Discovery Service within the University of Witwatersrand’s e-library resources to augment the GS search and to widen the scope and depth of our search. Our search was limited to papers published in English between 1990 and 2019 covering climate change and variability dynamics, climate modelling and hydrological modelling covering briefly the global, continental, regional perspectives and then more extensively the Zimbabwean context. The literature search inclusion and exclusion criteria are summarised in Table 1. Thematic analysis adapting and integrating the approaches of Perkins et al. [73] and Nichols et al. [74] was used in assessing the content of the selected journal articles and categorising them according the study keywords and their dominant/predominant focus area/theme, e.g. general climate trends study, climate impact and climate modelling. Climate impact studies were further categorised according to impact areas, e.g. agricultural impacts, livelihood impacts, ecological impacts, hydrological impacts and energy impacts. We also identified and categorised studies specifically integrating hydrological modelling and climate modelling leveraging geographic information systems (GIS) and remote sensing (RS) techniques. The various hydrological and climate modelling techniques/tools used in the selected studies were also assessed. General descriptive statistics (frequencies and proportions), Tables and Pie charts were used to present the findings of the study. Figure 1 summarises the main steps of our study methodology. However, it is important to note that some relevant published studies could have been missed probably due to poor indexing or publication in unrated online journals and databases. Furthermore, some studies covered more than one theme which meant that they had to be categorised in more than one group. Figure 2 is a map of the study area (Zimbabwe) showing the main water catchments, settlements and the hydrology (rivers and dams).

**Table 1:** Literature inclusion and exclusion criteria summary used to select articles covered in the review

Inclusion criteria	Exclusion criteria
Published, peer-reviewed academic journal articles on global, continental and regional scope on climate change and variability (trends) and hydrology	Unpublished, non-peer-reviewed materials
Published, peer-reviewed academic journal articles on climate change and variability, climate and hydrological modelling in general and specifically in Zimbabwe	News articles, Unpublished thesis, Unofficial reports, Blog sites materials
Peer-reviewed journal articles on climate impacts in general and local (Zimbabwe)	Old publication (> 15 years) for global, continental and regional scope
Peer-reviewed journal articles published in English language from 1990 (for Zimbabwe scope)	Non-English language publications
Published book sections/chapters, ebooks, Reports (used in discussion only)	General, non-scientific reports

## Results and discussion

### Climate change/variability

#### Global and regional climate change/variability studies: a brief overview

Climate change refers to a statistically significant variation in either the mean state of the climate or in its variability, persisting for an extended period (typically decades or longer) due to natural internal processes or external forcings, or persistent anthropogenic changes in the composition of the atmosphere or in land use [75]. Most scientists have, however, settled to use the term climate change to refer primarily to observed and predicted changes mainly as a result of human activities [53, 76–78], though others suggest that climate changes are a result of natural cycles [3]. Over the years, the debate has evolved to include populist ideologies charged with political undertones [79–83], while some have presented alternative views in what has been termed the ‘climate change hiatus’ where scientists are beginning to re-interrogate if the temporary slowdown in the global average surface temperature warming trend observed between 1998 and 2013 is a genuine slow down or a redistribution of energy in the earth system [45, 84, 85]. Climate variability on the other hand has been defined as variations in the mean state and other statistics (such as standard deviations and the occurrence of extremes) of the climate on all temporal and spatial scales beyond that of individual weather events due to natural internal processes within the climate system (i.e. internal variability), or variations in natural or anthropogenic external forcing (that is external variability) [75].

Findings by the Inter-governmental Panel on Climate Change (IPCC) experts [86–88] and other studies such as Scholes et al. [31], Zachos et al. [2] and Stocker et al. [89] indicate that the earth's climate has experienced complex evolution marked by periodic and anomalous variability both at global and regional scales with diverse impacts on populations throughout-time. Such changing climatic patterns have been linked with various extreme events or phenomena such as droughts and floods [13, 42, 54, 57, 90]. This notion is also buttressed in a review of observed (1900–2000) and possible future (2000–2100) climatic conditions across Africa by Hulme et al. [30] which concluded that the climate of Africa is warmer than it was 100 year ago with some regions experiencing substantial inter-annual and multi-decadal rainfall variations with dramatic impacts on both the environment and some economies. Impacts of anthropogenic processes on the global carbon cycle and the resultant greenhouse effect have been acknowledged as directly linked to global and regional climatic systems perturbations with the same devastating effects on numerous vulnerable communities around the world, for example [29, 75, 88, 91, 92]. To mitigate against such impacts, 197 countries signed the 2015 Paris Climate Agreement in which signatories agreed to a goal of holding global temperatures well below 2 °C above the pre-industrial levels and to pursue efforts to limit it to 1.5 °C [88]. The IPCC further emphasised a dire need for drastic global action [93] to achieve this in light of a narrow window period of up to 2030 to stem catastrophic climate change projected by scientists such as Miller, Croft [94]. However, such global climate change governance efforts have not been without major drawbacks as highlighted by the withdrawal of the United States of America (USA) in 2017 from the Paris Accord citing unfairness of the agreement and possible threats to US economic interests [95–97]. Furthermore, it is worth mentioning that the recent SARS-CoV-2 (COVID-19) pandemic has also brought in a new dimension into the existing global climate change research and governance discourse [98–100] though this is not within of the scope of this review.

Climate vulnerability describes the degree to which a system is susceptible to, and unable to cope with, adverse effects of climate change and variability and extremes [75]. It is a function of climate sensitivity and adaptive capacity of communities and natural systems [101]. Climate change increases frequency and intensity of extreme events such as storms, droughts and wildfires which impact on the global food supply, displace communities, and disrupt livelihoods thus increasing the risk of conflict, hunger and poverty [102]. Numerous climate impacts studies covering vulnerabilities and adaptation such as [15, 103–106] have been done and the general consensus is that climate change and variability present serious vulnerability challenges (as earlier alluded to) in most regions globally. Within the semi-arid regions in Southern Africa, challenges such as crop failure, land degradation and deforestation are prominent considering their ecosystems and agriculture depend on rainfall for their primary production. In this regard, researchers such as Berrang-Ford et al. [107] have explored human climate adaptation actions while others such as Anwar et al. [108], Lennard et al. [109] and Reddy [13] have in this regard researched on modalities of developing frameworks for characterising and understanding community adaptation capacities to climatic variability and change *vis-à-vis* the spatio-temporal dynamics of climatic events such as the El Niño-Southern Oscillation (ENSO). Among other conclusions drawn, all these have revealed a need for pragmatic policy development buttressed by sound scientific evidence to guide mitigation and adaptation strategies especially in developing countries such as Zimbabwe [34].

#### Climatic change and variability studies in Zimbabwe

Climate in Zimbabwe is highly variable [110] and thus the country (with its limited coping capacity) is considered highly vulnerable to climate change and variability impacts like most developing countries in Africa, e.g. [86, 91, 111–113]. In light of this, notable response initiatives have been taken by the Government of Zimbabwe (GoZ) in line with SADC climate policy directions. These include the adoption of a National Climate Policy (NCP) augmented by a National Climate Change Response Strategy (NCCRS) and the setting up of a dedicated National Climate Change Management Department under the then Ministry of Environment, Water and Climate in 2013 (now Ministry of Environment, Tourism and Hospitality Industry). One of the six core objectives of the NCP was to strengthen climate research and modelling and promote relevant home-grown solutions to address the challenges of climate change [71]. Furthermore, the GoZ in partnership with the United Nations Development Programme (UNDP) implemented the National Adaptation Plan (NAP) in the year 2017 which was aimed at analysing the country’s short- and long-term climate risks and adaptation options so as to help feed-into the country’s NCP and NCCRS up-scaling of climate resilient development initiatives. In 2018, the GoZ set up and launched the Zimbabwe National Geospatial and Space Agency (ZINGSA) under the Ministry of Higher and Tertiary Education, Innovation, Science and Technology Development with one of its mandate being to leverage exploitation of earth observation and geospatial technology in advancing climate science research among other focus areas. These developments have come against the backdrop of the GoZ launching the Zimbabwe Centre for High Performance Computing (ZCHPC) in 2015 aiming at availing supercomputing capabilities to researchers in various field of science such as meteorology (for example in numerical weather prediction/forecasting) and earth-system modelling in the country [114]. Despite these positive developments, not much research has been undertaken to expand knowledge and present updated scientific information on the evolution of past and future climatic conditions in Zimbabwe so as to buttress and enhance the country’s resolve/focus in achieving the Sustainable Development Goals (SDGs) such as SDG 1, 2 and 13 (that is No poverty, Zero hunger and Climate action, respectively). A synergy in all these developments, i.e. the ZINGSA, the ZCHPC and other relevant state agencies is critical to realise overall national climate objectives.

**Fig. 3:**
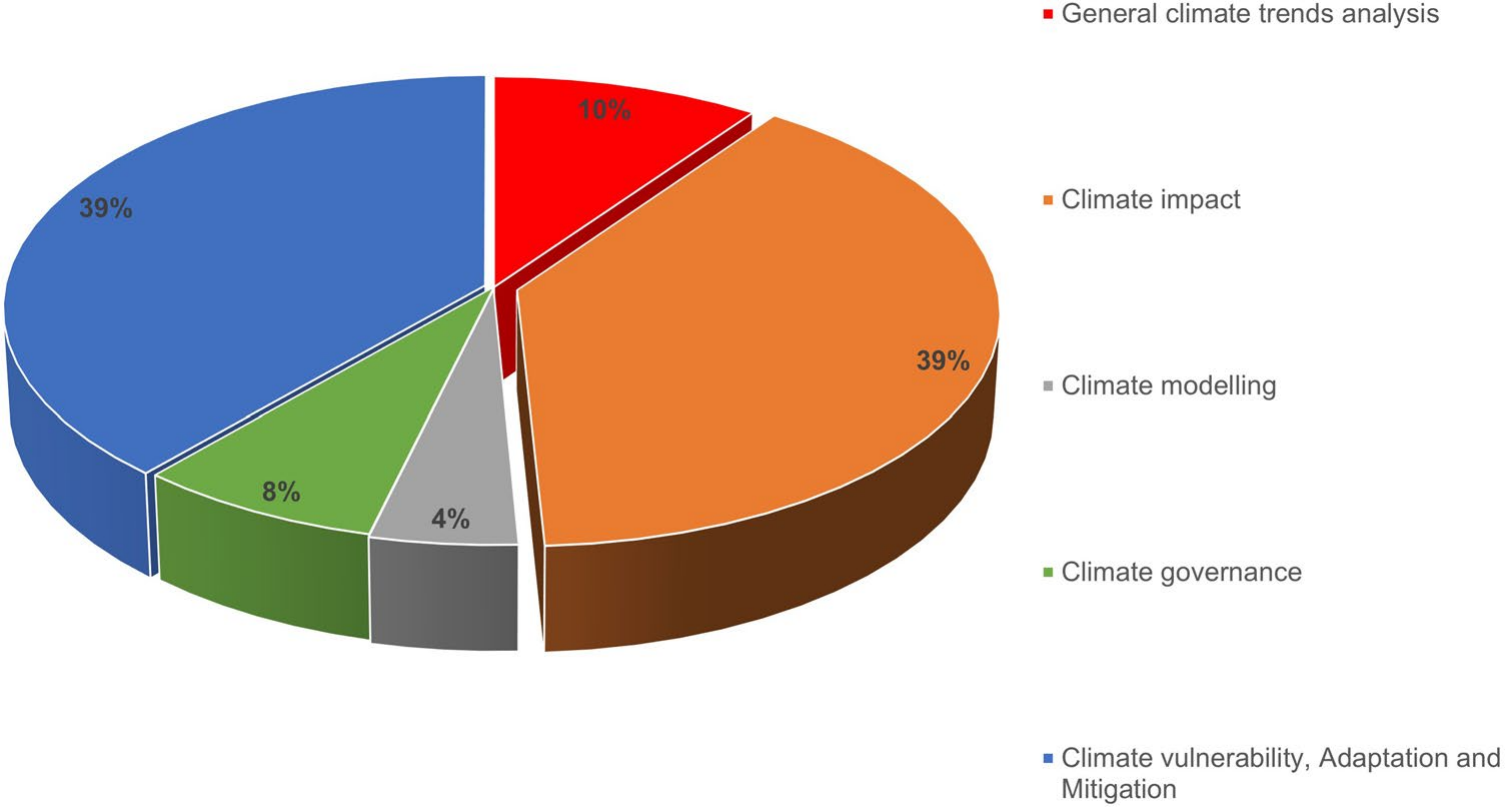
Graph showing proportions (%) of the various climate study themes (categories) covered in Zimbabwe in the past 29 years (*n* = 107). Climate impact studies are predominant

Figure 3 shows proportions of the five main identified thematic groups/categories from the climate studies covered under this review while Table 2 gives some examples of the 107 prominent climate studies undertaken in the past 29 years in Zimbabwe under each category. Researchers from Zimbabwean-based research institutions/organisations directly and in collaboration have over this time period contributed approximately 70% of the studies reviewed herein. The rest are contributions come from researchers affiliated to research institutions outside of Zimbabwe. Climate impact and climate vulnerability, adaptation and mitigation studies are the co-predominant categories of all the studies reviewed (each with 39%), while climate modelling is the least covered theme (9%) followed by climate governance studies (8%). These results reveal a dearth of scientific knowledge primarily within the themes of climate modelling, climate governance and general climatic trends, respectively, in Zimbabwe. Results show a publication rate of 3.7 (approximately 4) journal article publications per year over the study period which shows relatively low research output on climate science in Zimbabwe, thus, further demonstrating a knowledge gap in this regard.

**Table 2:** Summary table showing climate study categorisation, examples and statistics for each category

Study category	Frequency	%	Examples of studies
General climate trends analysis	13	10	(Unganai [115])*, (Unganai [129])*, (Unganai and Mason [120])*, (Williams et al. [339])*, (Mazvimavi [116]), (Nyoni et al. [293]), (Love et al. [330]), (Mushore et al. [143]), (Sibanda [341]), (Mamombe et al. [119])
Climate Impact	52	39	(Booth et al. [333]), (Unganai [117]), (Salewicz [249]), (Kristensen et al. [191]), (Corbett and Carter [141])*, (Makadho [199]), (Hartman et al. [342])*, (Chemura et al. [331]), (Matarira et al. [325])*, (Nyanganyura [246]), (Pilossof [237]), (Ebi et al. [236])*, (Brown et al. [130]), (Mutekwa [327])*, (Williams et al. [339])*, (Gwimbi [242]), (Mugandani et al. [343]), (Nyabako and Manzungu [231]), (Gwitira et al. [230]), (Chapungu and Nhamo [338]), (Ncube [235]), (Love et al. [330]), (Phillips et al. [344])*, (Manyeruke et al. [322]), (Davis and Hirji [253]), (Sango and Nhamo [198])*, (Sande et al. [22]), (Pedersen et al. [21]), (Beck and Bernauer [250]), (Yamba et al. [223]), (Kutywayo et al. [345]), (Torr and Hargrove [335]), (Svotwa et al. [324]), (Zinyengere et al. [328]), (Dube et al. [347]), (Bhatasara [287]), (Zinyemba et al. [369]), (Bossuet and Thierfelder [348]), (Magadza [349]), (Gunda et al. [336]), (Descheemaeker et al. [350])*, (Chikodzl and Mutowo [337]), (Utete et al. [234]), (Mamombe [119]), (Lord et al. [192])
Climate modelling	6	5	(Unganai [115])*, (Ebi et al. [236])*, (Mushore et al. [143]), (Chemura et al. [331])*, (Mashonjowa et al. [351]), (Masanganise et al. [323])
Climate governance	10	8	(Patt [326])*, (Patt and Gwata [197]), (Zinyengere et al. [328])*, (Gutsa [123]), (Mberego and Sanga-Ngoie [352]), (Ngwenya et al. [122]), (Bhatasara [70]), (Mubaya and Mafongoya [257])
Climate vulnerability, Adaptation and Mitigation	51	39	(Matarira et al. [325]), (Matarira and Mwamuka [353]), (Unganai [329])*, (Patt [326])*, (Thierfelder and Wall [121]), (Rurinda et al. [354]), (Chifamba and Mashavira [355])*, (Gwimbi [242]), (Mudombi-Rusinamhodzi et al. [356]), (Gwenzi et al. [251]), (Nhemachena et al. [357]), (Soropa et al. [258]), (Dube et al. [346]), (Descheemaeker et al. [350])*, (Chanza [370]), (Manyani et al. [243]), (Moyo [125]), (Mbereko et al. [358]), (Musarandega et al. [359]), (Mubaya et al., [257])*, (Chanza et al. [360]), (Mushawemhuka et al. [361]), (Jiri et al. [362]), (Mugambiwa [260]), (Katsaruware-Chapoto et al. [364]), (Mutandwa et al. [365]), (Jiri et al. [363]), (Mugambiwa [260]), (Simba et al. [366]), (Nyahunda and Tirivangasi [367]), (Nyahunda et al. [368])

NB, Twenty-five (25) of the studies marked with an asterisk (*) fall within at least two categories considering their scope)

One prominent study shown in Table 2 that exclusively explored climate conditions in Zimbabwe is by Unganai [115]. This study revealed that over a 93 year period from the 1900s, daytime temperatures in Zimbabwe rose by about 0.8 °C, translating to a 0.08 °C rise per decade while precipitation was observed to have declined by up to 10% on average over the same period, which is about 1% per decade. These findings, however, were rebutted by Mazvimavi [116] in his study covering 40 rainfall stations across all the rainfall regions of Zimbabwe for periods 1892–1941 and 1942 to 2000. Mazvimavi [116] concluded that the purported climate change effects were not statistically significant within the time series of total seasonal and annual rainfall in Zimbabwe, arguing that the findings of declining rainfall by Unganai [115] were likely due to the presence of multi-decadal variability characterised by combining years with above and below average rainfall. This contrast between two prominent climate researchers presents a need for interrogation of data with new/updated techniques to bring better clarity with regard to past climatic conditions/trends in Zimbabwe. Since then, no known, published follow-up study has been done to build upon the existing knowledge in this regard.

On rainfall variability, studies have revealed that inter-annual rainfall variability in the country are largely influenced by external forcing of a near-global or hemispheric origin such the ENSO, the Inter-tropical Convergence Zone (ITCZ) to the North and the westerly cloud-bands to the south rather than regional or local-scale factors [117–119]. On long-term predictability of rainfall trends, Unganai, Mason [120] indicate that approximately 70% of the total summer rainfall variance in Zimbabwe is potentially predictable at long range due to due to a high climate signal to noise ration especially in the in the eastern, central, and western parts of the country. Over the years, climate-rainfall research has advanced to explore the teleconnectivity between summer rainfall patterns in Zimbabwe and sea-surface temperatures (SST), the Southern Oscillation Index (SOI), the Quasi-biennial Oscillation (QBO), Outgoing Longwave Radiation (OLR) and wind [32]. Nangombe et al. [121], for example, concluded that there are strong correlations between severe droughts and circulation patterns and weather systems in the Indian Ocean and Equatorial Pacific Ocean such as the ENSO SOI, the QBO and the Luni-solar tide at 20, 12.5, 3.2, and 2.7 year cycles. These studies have revealed the possibility of predicting drought occurrences using these established relationships. However, this knowledge is rather outdated and has not been fully utilised by decision makers in Zimbabwe for enhanced drought and other climate impact mitigation fore-planning [122, 123]. This is evidenced by poor preparation and the resultant recurrent adverse impacts experienced when such events occur.

Within the general climate studies reviewed in this paper, the predominant area of focus has been understanding the rainfall dynamics [120, 121, 124, 125] with less attention on temperature and other climatic parameters such as evaporation, humidity and solar radiation. Limited research on these other climatic parameters could be attributed to limited access to good quality data as revealed by Dlamini et al. [126]. However, some studies that have looked at other climatic parameters such a temperature and evapotranspiration [30, 110, 127, 128] had limited detail that could not give a comprehensive picture of the dynamics of these parameters in space and time in Zimbabwe. For example, though Unganai [129] concluded a net warming of + 0.3 to + 0.5 °C between 1897 and 1993, he could not attribute the observed warming trend to inherent climate variability though similar trends have been later related to climate change by Brown et al. [130] and Watson et al. [131]. Some of these studies have presented the climate dynamics in Zimbabwe in a general sense considering that the studies had a regional scope of coverage (covered Southern Africa) [31, 127, 132, 133]. Furthermore, studies such as Matarira and Jury [134] had limited temporal resolution in their assessments since they used cross-sectional study designs and thus missed exploring the multi-temporal aspects of the climatic conditions in Zimbabwe. In other words, there is a need to build on this existing knowledge through longitudinal studies to capture a more recent picture of multi-temporal climatic trends in Zimbabwe.

While advances in climate research have seen the move towards the use of GIS and RS/Earth observation (EO) technology to (1) augment climate data series, and (2) assist in better and advanced analyses of climate dynamics in space and time globally [135–138] and in Southern African [139, 140], progress in this direction has been limited in Zimbabwe. Only 8% of the reviewed studies in Zimbabwe over the past 29 years have directly and indirectly applied these tools and techniques at varying spatial and temporal scales. Examples include an assessment of inter‐seasonal rainfall variability in Zimbabwe using GIS by Corbett, Carter [141], spatial characterisation of summer rainfall Zimbabwe by Unganai, Mason [118], spatio-temporal analysis of climate-inter-annual malaria incidence [142], and exploring local climate zones-land surface temperature interlinkages using remotely sensed data [143]. This reveals a knowledge gap (of limited use of geospatial tools in climate research) which could be worsened by limited availability of quality *in situ* climatic data such as rainfall and temperature measurements. Furthermore, where such data is available, often it is incomplete due to poor distribution and investment in necessary infrastructure/facilities to observe important climatic phenomena. Other data access challenges relate to (1) inaccessibility due to bureaucratic red-tapes and prohibitive costs for long-term climatic datasets charged by government agencies such as the Meteorological Department, (2) inconsistent and poor spatial coverage which often renders it of limited use in climate research in the country. This is also confirmed by Gumindoga et al. [144] who noted that historic temperature and rainfall data for Zimbabwe is incomplete and often costly to purchase thus a limiting factor in climate research in general. In light of such limitations, researchers such as Chikodzi [145], Dlamini et al. [126], Kamusoko, Aniya [146] and Mpala et al*.* [147] have exploited freely available remotely sensed climatic and other datasets to overcome these challenge, notwithstanding the inherent spatial and in some instances temporal resolution limitations of using these datasets at a local scale. The main advantages of using remotely sensed datasets include their availability and access at low to no cost (freely available online) and their ability to give parameter measurements in otherwise inaccessible and ungauged areas at a wider spatial coverage compared to in situ measurement techniques. Disadvantages of remotely sensed data relate to spectral mixing due to low or course spatial resolution, limited temporal resolution which tend to limit time series analysis in some instances and no spatial coverage due to remote sensing satellites’ limited repeat cycles. Furthermore, very high purchase costs of higher resolution datasets tend to be prohibitive or limit access and use in developing countries.

### Climate modelling studies

#### A brief global and regional overview

Climate modelling science is a highly active field of research with rapid advancements in knowledge marked and driven by rapid developments in the tools and techniques (models) used in this domain. Two main types of models, i.e. Global Climate Models (GCMs) and Regional Climate Models (RCMs), are used in climate modelling studies. GCMs are numerical tools/models representing physical processes in the atmosphere, ocean, cryosphere and land surface used for simulating the response of the global climate system to increasing greenhouse gas concentrations [91]. Examples of GCMs include the Hadley Centre Coupled Model, version 3 (HadCM3), the Commonwealth Scientific and Industrial Research Organisation Mark 3 (CSIRO Mk3) GCM [127, 148], the Geophysical Fluid Dynamics Laboratory Climate Model version 2.5 (GFDL CM2.5) [149] and the Model for Interdisciplinary Research on Climate–Earth Systems Model (MIROC-ESM) [150] and the more recent Hadley Centre Global Environment Model 3–Global Coupled version 3.1 (HadGEM3-GC3.1) [151]. Other variable resolution GCMs such as the Conformal-Cubic Atmospheric Model (CCAM) of the CSIRO have also been developed for regional climate and weather research [152, 153] and have been applied at different scales globally. Over the years, the GCMs have been used to improve our understanding of how climate systems work, to forecast the drivers of climate change, improve estimates of climate sensitivity and to predict future climatic conditions and impacts, e.g. [46, 89, 154, 155]. Advances in this domain have seen the progression from Atmosphere-only GCMS (AGCMs), to Coupled Atmosphere–Ocean models (AOGCM) and fully coupled earth system models (ESM) in an attempt to improve the statistical confidence in the GCM outputs. Thus, the emergence of AOGCMs has allowed for more reliable projections of climate at various spatial and temporal scales [155–157]. This has been realised in light of the well appreciated inherent uncertainties and weaknesses associated with the use of such models. For example, Motesharrei et al. [158] argued that two-way feedbacks are missing from most climate models and other critical socio-economic variables such as inequality, consumption, and population are often inadequately modelled hence increasing uncertainty in outputs. Fowler et al. [159] further emphasise that GCMs have relatively coarse resolutions and hence are unable to resolve significant sub-grid scale features such as land use and land cover (LULC) and topography, thus limiting their accuracy and application at a local scale. To this end, the IPCC and other climate scientists have progressed to implement and develop the Coupled Model Intercomparison Project (CMIP) with the latest being CMIP6 [160]. In CMIP6, various ensembles of GCMs have been run collectively and results compared in an attempt to understand how the global climate will respond to future scenarios of increasing/decreasing anthropogenic radiative forcing relative to present‐day climate conditions [157, 161, 162]. For example, Andrews et al. [163] recently ran simulations using the HadGEM3-GC3.1 for CMIP6, testing climatic responses to historical forcings such as solar irradiance, ozone concentrations, greenhouse gases, land‐use changes, and aerosols compared results to observational data.

To resolve the shortcomings of GCMs, downscaling techniques [164–166] have been used to develop finer resolution Regional Climatic Models with varying levels of accuracies at sub-grid scale with higher statistical validity and reduced biases compared to GCM simulation outputs. Examples of such RCMs include the Consortium for Small-Scale Modelling and Regional Climate Model (COSMO-CLM), Regional Climate Model version 4 (RegCM4), and the Providing Regional Climates for Impacts Studies (PRECIS) model. Because of their higher resolution (compared to GCMs), RCM data have been widely used in numerous impact studies as input in hydrological models for example, in an attempt to assess the variability of hydrological responses due to past, present and future climate change scenarios [133, 167–169]. Furthermore, to drive and coordinate active research in both dynamic and statistical regional climate downscaling (RCD) techniques of GCMs so as to provide higher-resolution climate information at regional level, the World Climate Research Programme (WCRP) has run the Coordinated Regional Climate Downscaling Experiment (CORDEX) [170]. The CORDEX has, over the years, allowed for an objective assessment and intercomparison of various RCD techniques, i.e. an evaluation of their performance, illustration of benefits and shortcomings of different approaches, thus providing a more solid scientific basis for impact assessments and other uses of downscaled climate simulations. To this end, high resolution regional climate simulations of the CORDEX CORE activity are now available covering all major inhabited areas of the world at a resolution of 25 km including Africa (Domain: Region 5).

Numerous climate modelling studies have been done both at global and regional scale in an effort to better understand the past, present and future climate dynamics in space and time. While significant progress has been realised in global climate modelling science, e.g. [5, 163, 171–174], there has been relatively less work published for Africa in the same regard [30] and let alone at local (country) level. Some countries such as South Africa have been leading and have made considerable strides in climate modelling research and actively contributing to the IPCC working groups and the CORDEX-Africa for example. GCMs such as the Canadian Climate Centre Model (CCCM) and GFDL-3 have simulated changes of plus 2 to 4 °C increases in mean surface air temperature across Southern Africa under doubled atmospheric carbon dioxide scenarios showing over and underestimations when validated with observed data over local areas [128]. Other models that have been applied in Africa include the HadCM3, Parallel Climate Model (PCM) and the Coupled Global Climate Model (CGCM3) [175] showing varying simulation outputs with limited local use considering their inherent uncertainties related to forcings and horizontal biases as discussed by Arora [176].

On the contrary, downscaled RCMs have demonstrated more competence in simulating local climatic conditions compared to GCMs [165, 177–180] though the contradictions and parameter over and underestimation of rainfall and temperature scenarios still persists when model outputs are compared [155, 181, 182]. For example, RCMs have shown to successfully simulate future projection of droughts in Southern Africa [179]; predict seasonal and regional climatic scenarios [180]; and project an annual-averaged temperature rise at about 1.5 times the global rate of temperature increase in the African subtropics during the twenty-first century [183]. The general consensus, however, among climate scientists is that projections of future climate change are restricted to assumptions of climate forcing, limited by shortcomings of the climate models used and inherently subject to internal variability when considering specific periods [165, 184–186]. This justifies the need for sustained research in regional climate downscaling research as supported in CORDEX framework. Resultantly, numerous studies, e.g. [167, 177, 187–190], have been undertaken in Africa and Southern Africa under the CORDEX showing remarkable advancements with more accurate and region-relevant results. Details of the findings of these studies are outside the scope of this review.

#### Climate modelling studies in Zimbabwe

Despite the advances in climate modelling science globally and regionally as earlier alluded to, the scope of climate modelling research in Zimbabwe has been rather limited. Of the 107 prominent climate studies done in Zimbabwe (covered in this review), only 8% of these directly and indirectly involved climate modelling at some level as earlier shown in Fig. 1. The studies covered climate modelling in relation to aspects such as disease vector distribution, e.g. Kristensen et al. [191], Lord et al. [192] and Gwitira et al. [193], climate impacts on hydrological systems [194–196], agricultural productivity [197] and urban environments and natural ecosystems [143, 195, 198] and general prediction of future climatic conditions [115]. Unganai [128] used two GCMs (the GFDL and the CCCM) to simulate future climate conditions for Zimbabwe using a doubled carbon dioxide concentration forcing and concluded that the models were inefficient in predicting the magnitude of precipitation change for example. Similarly, Makadho [199] used the same two GCMs to assess potential impacts of climate change on maize production while Matarira and Mwamuka [200] used the Goddard Institute of Space Studies (GISS) model to assess forest vulnerability to climate change. Simulated maize yields decreased considerably under dryland conditions based on the climate change scenarios largely due to shorter growing seasons driven by increased temperatures (ibid). Matarira et al. [201], on the other hand, tested a combined crop model (CERES-Maize) with climate scenarios derived from two GCMs which showed that future low rainfall and high temperature will threaten agricultural production in Zimbabwe. In their stable malaria transmission study, Ebi et al. [202] tested four GCMs, i.e. the CCCM, the United Kingdom Meteorological Office (UKMO) model, and the GISS model to simulate and relate future climatic scenarios to malaria transmission. They concluded that changes in temperature and precipitation due to climate change could alter the spatial distribution of malaria in Zimbabwe, with previously unsuitable areas of dense human population becoming suitable for transmission.

From all these reviewed modelling studies, most used GCM simulations directly, which could have had negative implications on the accuracy of their findings due to the known inherent limitations of GCMs application at a local level. A few exceptions such as Pedersen et al. [21] and Moyo, Nangombe [133] attempted to use RCMs and applied downscaling techniques to generate more accurate climate simulations from GCMs for their studies. For example, Makuvaro [203] used Statistical and Regional dynamic Downscaling of Extremes for European regions (STARDEX) to come up with downscaled local scenarios for his study. Overall, it is emerging that most of these studies have used GCMs and RCMs (downscaled or directly) with limited regard of inherent limitations of these different models implying possible inaccuracies in some of the findings and conclusions of these studies. This is in light of the fact that most models are developed to be more region specific and transferring them to or applying them in other regions of the world will give inaccurate results by the inherent design of the models. Furthermore, considering the advances in revision and/or improvements on old climate models and the development of new, better and region specific models, e.g. under the CORDEX programme [167, 168], it is apparent that there is a need to revisit and advance climate modelling science in Zimbabwe. This means testing new, and advanced region specific downscaled RCMs or GCMs so as to help fill the earlier discussed climate science knowledge gaps in the country. We also found that no known study has been done to comparatively assess the competence of the more recent RCMs and GCMs in simulating local climate over Zimbabwe. Furthermore, there has been no development of contextual optimization of RCMs to improve their skill in reproducing local climatic characteristics building on the research work covering Southern Africa by scientists such as Engelbrecht et al. [204], Dosio [205] and Abiodun et al. [206]. Such research advances and the ensuing results could help inform more contextual national climate adaptation and mitigation policy appraisal and response strategy development by the GoZ as earlier discussed.

### Climate change/variability impact studies

#### A general global overview

A number of climate scholars have explored the impacts of climate change and/or variability on various natural and human systems [54, 75, 91, 184, 207–210] and the results indicate heightened community vulnerabilities [12, 104, 106, 211, 212] at a global, regional and local scale. Amongst other noted impacts, most of the studies have shown that climate change has an overall negative impact on hydrological systems in the world [213–215]. Arnell [6] for example, noted reduced runoff in the Mediterranean, Central and Southern America, and Southern Africa and increased evaporation in some areas [140, 216]. In Southern Africa, climate change has also been linked to the El Niño–Southern Oscillation (ENSO) induced droughts, e.g. [132, 168, 178, 217–219] with devastating effects on communities and the environment in general. Several studies have quantified the extent of impacts and their dynamics (in space and time) on water resources [5–9]; food security [12–14, 220]; ecosystems, e.g. [17, 209, 221, 222]; energy, e.g. [146, 223–226]; and human health [21, 22, 227]. All these studies have revealed that climate change is a very significant factor to consider in holistic planning for community resilience and adaption for sustainable development, and more importantly in African developing countries such as Zimbabwe. Considering the intricate coupling of the human and natural systems, most of these studies have used diverse advanced methods in an attempt to understand the climate change dynamics *vis-à-vis* all the earlier mentioned factors. Of note has been the widespread use of climate model simulations (from both GCMs and RCMs) in climate impact models to explore how natural and human systems may be affected by climate change [6, 155]. That is, the climate simulations have been used in integrated climate change impacts assessments notwithstanding the limitations of under and overestimating some climate extremes impacts revealed by Schewe et al. [228].

While these impacts are well acknowledged to be more devastating in vulnerable communities in developing countries due to their weak institutional arrangements and policies for resilience and adaptation [103], climate science research still lags behind in most of these countries [16, 76, 229]. This has heightened future climate vulnerability due to limited scientific knowledge to guide pragmatic policy development and strategies for adaptation and resilience.

#### Climatic change impact studies in Zimbabwe

**Fig.4:**
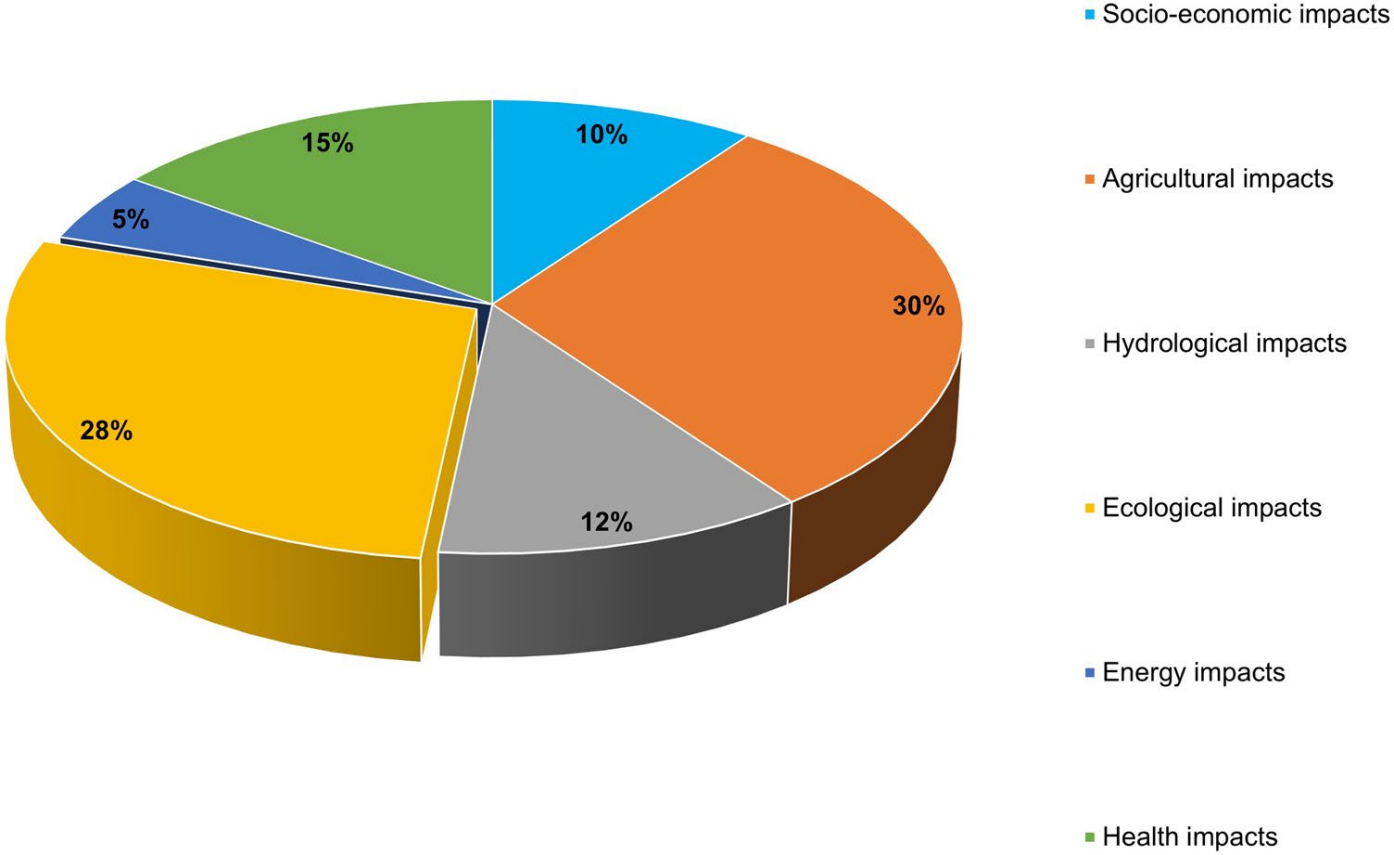
Chart showing proportions (%) of the various climate impact study themes covered in climate studies in Zimbabwe in the past 29 years. Ecological and agricultural impacts are the predominant themes covered by the impact studies (*n* = 52)

In this review, 52 of the 107 (39%) climate studies done in Zimbabwe over the past 29 years were found to be climate impact studies as earlier alluded to. Table 3 and Fig. 4 show thematic summaries of our findings in this regard. The emerging themes/categories covered by these studies ranged from climate agricultural impacts [199, 230–232]; socio-economic impacts [130, 233, 234]; ecological impacts, hydrological impacts, e.g. [117, 194, 235]; energy impacts [224]; and health impacts, e.g. [236, 237]. Agricultural impacts, ecological impacts and health impacts studies were found to be the three top categories of impact studies while energy impacts studies were the least covered category over the past 29 years in Zimbabwe as shown in Fig. 4. The scope of coverage of these studies ranged from national through district, ward to catchment level. This basically revealed limited and, in some instances, outdated scientific knowledge in the least covered categories (i.e. socio-economic, energy and hydrological impacts of climate change). With limited research output informing climate impact on energy, it may imply that planning for energy security in the face of climate risks could be a major contributing factor to the persistent energy problems that the country has been grappling with. The energy crisis has compounded over the years climaxing with the recent (2019) near total shutdown of the country’s main hydro-electricity power source, i.e. the Kariba South Power station due to poor rains and the resultant low inflows into the Kariba reservoir [238].

**Table 3:** Summary table showing climate impact studies categories and study examples done in Zimbabwe from 1990 to 2019 (*n* = 52)

Study theme	Frequency	Examples of studies
Socio-economic impacts	6	(Matarira and Mwamuka [201]), (Dube et al. [255]), (Brown et al. [130]), (Manyeruke et al. [322]), (Utete et al. [234])
Agricultural impacts	18	(Corbett and Carter [141]), (Makadho [199]), (Gwimbi [242]), (Matarira et al. [325]), (Masanganise et al. [323]), (Svotwa et al. [324]), (Patt and Gwata [197]), (Nyabako and Manzungu [231]), (Mutekwa [327]), (Zinyengere et al. [328]), (Unganai [329])
Hydrological impacts	7	(Unganai [117]), (Salewicz [249])*, (Davis and Hirji [253]), (Love et al. [196]), (Chemura et al. [331]), (Mamombe [119])
Ecological impacts	17	(Nyanganyura [246]), (Gwitira et al. [230]), (Marshall 332), (Booth et al. [333]), (Sango et al. [198]), (Pilossof [237])*, (Gandiwa and Zisadza [334]), (Sango and Nhamo [198]), (Magadza [349]), (Pedersen et al. [21])*, (Chikodzl and Mutowo [337]), (Chapungu and Nhamo [338]), (Gwitira et al. [193])*, (Matawa et al. 2013)
Energy impacts	3	(Salewicz [249])*, (Spalding-Fecher et al. [224]), (Yamba et al. [223])
Health impacts	9	(Williams et al. [339]), (Ebi et al. [236]), (Gwitira et al. [193])*, (Pilossof [237])*, (Pedersen et al. [21])*, (Torr and Hargrove [335]), (Gunda et al. [336]), (Kristensen et al. [191])

NB, Eight (8) of the studies marked with an asterisk (*) fall within at least two categories considering their scope

Some of the emerging general conclusions from the reviewed impact studies are presented hereon. Climate-agricultural impact studies generally revealed that smallholder agricultural production is significantly constrained by high temperature and low rainfall in Zimbabwe [233] and that climate change has compounded Zimbabwean peasant farmers’ climate vulnerability, e.g. to drought hence food insecurity and poverty [239]. This has necessitated pragmatic adaptive management of agro-biodiversity to ensure equitable sharing of resources in the face of climate change and uncertainties as suggested by Masocha et al. [240]. Given that smallholder and subsistence farmers are highly vulnerable to climate change [241], there is need for deliberate investment in climate adaptation strategies and clear policies on irrigation and early warning systems to bolster the farmers’ climate resilience [141, 230] in line with the Southern African Development Community (SADC) Climate Change Adaptation Strategy [103]. Crop production has also been related to climatic conditions in some of these studies. For example, cotton production levels were noted to have declined as precipitation decreased and temperatures increased in Gokwe district of Zimbabwe [242], while maize productivity has been projected to decrease in response to various global climate change scenarios [231, 243]. The ENSO has been successfully linked to rainfall, drought and maize yield, e.g. [231, 242, 244–247] and livestock productivity [248]. Within agro-ecology impacts, Mugandani et al. [135] and Chingombe et al. [245] agree that the main food production regions of Zimbabwe (regions 1 and 2) are likely to decrease in size due to climate change and variability pointing to possible reduction in food production and food security hence the need to have commensurate mitigation measures to avert potential negative impacts.

As earlier presented, there are other emerging themes related to ecosystem impacts [21]. Matarira and Mwamuka [200] in their modelling study projected a 17 to 18% land area shift from subtropical thorn woodland and subtropical dry forest to tropical very dry forest under a modelled climate scenario of reduced precipitation and an increase in ambient temperatures. Climate change/variability has been shown to be a current and future threat to energy security in Zimbabwe, i.e. hydroelectric power potential will be reduced in all existing and proposed hydroelectric power schemes due to climate change and increasing water demand, e.g. [131, 223, 249, 250]. The energy-climate link is already evident as earlier discussed in the recent Kariba power station situation. With regard to public health, literature reviewed in this study indicate negative impacts/trends, e.g. results from a climate suitability for stable malaria transmission in Zimbabwe under different climate change scenarios by Ebi et al. [202] suggest that changes in temperature and precipitation could alter the spatial distribution of malaria in Zimbabwe, with previously malaria unsuitable areas of dense human population such as Bulawayo becoming suitable for transmission. Gwitira et al. [193] and Ebi et al. [236] have all concluded that climatic factors such as temperature and annual precipitation are critical factors intricately linked to current and possibly future changes in distribution of malaria in Zimbabwe. Other studies are indicating changes in abundance and distribution of tsetse flies, suggesting possible redistribution of African trypanosomiasis (sleeping sickness) incidence in Zimbabwe in the future due to climate change [237]. Table 4 presents some of the projected climate impacts by sector for year 2080. Of note is that projections indicate worsening of negative impacts in almost all the sectors under consideration. For example, runoff is projected to decrease significantly within major catchments such as the Save and the Mzingwane with wide-ranging consequences for resident communities and in the face of high vulnerability and low resilience.

**Table 4:** Projected climate change impacts by sector in Zimbabwe. (Adopted from [251])

	Sector	Projected climate change impacts
1	General	Predicted warming of around 2degrees Celsius by 2080 Present southwest-northeast-east rainfall gradient will become steeper
2	Agriculture	General vulnerability of communal agriculture to climate change and variability Generally, maize suitable areas will decrease by 2080, while cotton and sorghum suitable areas will increase by 2080 In the south western parts of the country, sorghum and maize will become increasingly vulnerable to climate change while cotton will become less vulnerable In the north central and eastern parts of the country, maize, sorghum and cotton will become less vulnerable
3	Water	Overall, surface water resources are projected to be reduced significantly by 2080 irrespective of the scenario used North eastern and the eastern parts of Zimbabwe are predicted to experience a surplus in surface water while the western and southern parts of Zimbabwe are projected to experience a drying up Runoff will decrease significantly in the Mzingwane, Shashe, Nata, and Save catchments
4	Health	The area under high to extremely high malaria hazard will tend to increase by 2080 High malaria hazard will be concentrated in the low lying parts of the country including the Zambezi valley, and the South-east Lowveld Expected minimum pressure on plant diversity for best and worst case scenarios is 42%
5	Forestry and biodiversity	Net Primary Production (NPP) will decrease from the current average maximum of over 8 tonnes per hectare per year to just over 5 tonnes per hectare per year by 2080 translating to decreased rangeland carrying capacity for both livestock and wildlife
6	Human settlement	Any reduction in available water will lead to increased water scarcity thus impacting on livelihoods
7	Tourism	With decreasing rainfall and rising temperatures, significant declines in biodiversity are expected to occur in most parts of the country especially the western regions where most of the park estates are located Lower resilience of ecosystems to other global environmental changes

Some researchers in Zimbabwe such as Cobo et al. [252] and Davis, Hirji [253] corroborate the acknowledged conclusion by the IPCC [172] and Watson et al. [131] that observational records and climate projections provide abundant evidence that freshwater resources are vulnerable and will be strongly impacted by climate change, with wide-ranging consequences for human societies and ecosystems globally. Studies on climate impacts on water resources all indicate negative trends, i.e. reduction in water reservoir water levels, increased evaporation and surface and groundwater storage and hydro-electricity potential [134, 232, 235, 254] which are directly related to climate impacts on rainfall and temperature. Very few studies have focused on modelling the hydrological impacts of climate change and variability in Zimbabwe indicating a gap in knowledge in this regard. These are covered more extensively under hydrological modelling studies section of this review. Of note also is that 11% of all the climate impact studies reviewed here, directly and indirectly employed climate modelling and climate impacts modelling techniques in their methodologies notwithstanding the rapid advancements within this domain. This limited utilisation of these advanced climate modelling tools and techniques thus presents a need to advance research in this direction so as to expand knowledge and close such apparent gaps in climate impact studies in Zimbabwe for informed policy formulation and interventions. The same can be said about studies exploring climate impact on the tourism sector in Zimbabwe especially considering the revealed interlinkages between the aviation industry and climate change and the potential negative impacts such as threats to wildlife which may undermine future tourism operations and activities [255, 256].

Relative to these developments in methodologies in climate impact studies, there has been an increase in the integration of indigenous knowledge systems in climate research in Zimbabwe over the past decade. These studies have chattered a new frontier in climate research aimed at understanding aspects such as seasonal climate forecasts [257–259] and local climate adaptation practices and strategies, e.g. [243, 260–262]. Developments in this regard indicate a drive to leverage and streamline the existing local indigenous knowledge in the development of pragmatic, low-cost local climate interventions and mitigation strategies.

## Hydrological modelling

### Hydrological modelling studies: a general overview

Hydrological models are representative simplifications of complicated hydrological processes using mathematical means to demonstrate the principal elements of the processes, their combination and function as a comprehensive hydrologic system [263]. These hydrological models have been classified in various ways but Refsgaard and Knudsen [182] grouped them into three broad categories namely, (1) empirical black box models, (2) lumped conceptual models, and (3) distributed physically based system. Examples of these include the TANK model [264], Hydrologic Engineering Center's Hydraulic Modeling System (HEC-HMS) [265, 266], TOPMODEL, Système Hydrologique Européen (SHE), Soil and Water Assessment Tool (SWAT) [267, 268] and complex conceptual models such as MODified HYDROLOG (MODHYDROLOG) [269]. A review of the pros and cons of these models by Sivapalan et al. [270] and Jaiswal et al. [271] revealed that distributed physically based models have the advantage of accounting for spatial heterogeneities and provide detailed description of the hydrological processes in a catchment with limited demands of input data hence their widespread use in numerous hydrological studies [266, 272–276]. The same notion was confirmed by the World Meteorological Organization [181] in their inter-comparison of conceptual hydrological models for operational hydrological forecasting. Furthermore, considering that these models use parameters which are directly related to the physical characteristics of the river basins (e.g. topography, soil, LULC and geology) and account for spatial variability of meteorological conditions [182]. They have been very useful in studies advancing the understanding of changes in hydrological processes such as surface run-off [264, 269, 277, 278] and groundwater storage [7, 279] over space and time and simulating future hydrologic conditions.

### GIS and remote sensing in hydrological modelling

As alluded to earlier on, over the years, GIS and RS techniques have become indispensable in most state-of-the-art hydrological models premising on the extensive spatio-temporal data capture and analysis capabilities of these technologies. Three main applications of RS in hydrological modelling presented in numerous studies can be summarised as, (1) model parameter estimation with the aid of multi/hyper-spectral satellite data; (2) computation of historic monthly runoff using satellite data as input; and (3) real-time flood forecasting using radar rainfall measurements as input [280, 281]. In this regard, many researchers have used GIS and RS in hydrological modelling studies aimed at optimisation of catchment management in the Mediterranean regions [282], water resources management in India [283, 284], forest hydrology [285–287], assessing water quality *vis-à-vis* human activities in Korea [288], monitoring small dams in semi-arid regions [289, 290] and general parameterisation of hydrological models [273, 291–293]. GIS and RS have been noted to have a major advantage of accurately sizing and characterising catchments in rainfall-runoff modelling over and above the fact that analysis can be performed much faster, especially when there are complex mixtures of land use classes and different soil types [294]. In Africa, numerous studies [40, 286, 295–299] have also exploited the same tools and techniques to advance knowledge in this domain. This has been enhanced by improved and free access to valuable satellite earth observation data from various systems such as Meteorological satellites [300], and Tropical Rainfall Measuring Mission (TRMM) [139, 301]. All these studies indicate that globally, GIS and RS have become an almost indispensable part of hydrological modelling studies over the past decades.

To this end, in the face of considerable uncertainty in determining water availability/security relative to climate and land use-land cover changes which impact of hydrologic conditions, it is critical for water resources managers and decision makers to have a better and simplified understanding of past, present and ideally future hydrological processes dynamics/scenarios through sound water resources studies (which leverage GIS and Remote sensing technology) [302].

### Hydrological modelling studies in Zimbabwe

Of the 107 studies reviewed in this study, 7% directly and indirectly involved hydrological modelling, indicating very limited hydrological modelling research in Zimbabwe over the period under review. Hydrological modelling studies in Zimbabwe date back to 1986 when Knudsen et al. [303] tested the capability of the WATBAL model in simulating ungauged catchments using medium size dams in Zimbabwe. Another early study is by Vörösmarty, Moore [304] who used a simple catchment-scale model to simulate seasonal variation in discharge in the Zambezi river and how it might respond to climate and land use change. Though developments have been slow in the past 29 years, advances made thus far have seen shifts from use of simple statistical models to empirical-black box models, lumped conceptual models and more recently to coupled distributed physically based hydrological models such as SWAT and HecHMS. For example, Love et al. [305] used an empirical model (the Hydrologiska Byråns Vattenbalansavdelning (HBV) model) to simulate hydrological processes in the Northern Limpopo basin (Mzingwane catchment), while the HEC-HMS model has been successfully used in simulating run-off in the gauged and ungauged Upper Manyame sub-catchments of Zimbabwe [306, 307]. The same model has been applied by Gumindoga et al. [144] in modelling the water balance of the Lower Middle Zambezi Basin, successfully estimating the total inflows into the Cahora Bassa Dam and recommending ways of managing artificial floods in this basin. Mazvimavi [308] successfully demonstrated the application of two lumped conceptual models, i.e. the Thomas *abcd* model [309] and the Pitman model [310] to estimate catchment descriptors such as flow characteristics in 52 ungauged sub-catchments in all the seven main catchments of Zimbabwe. Other models that have been used in Zimbabwe include the Surface Energy Balance System (SEBS) Water Balance Model to determine actual evapotranspiration in the Upper Manyame catchment [311] and the TOPMODEL to simulate streamflow of Upper Save River catchment [312]. The flownet computational and modelling method [313] has been applied as well in groundwater recharge modelling within the Gwayi catchment. From these studies, it is apparent that the shift has been from lumped, empirical/mathematical-based models towards distributed physically based models in hydrological studies in Zimbabwe over the past 29 years.

A review of the scope of coverage of these studies revealed that all seven water catchments in Zimbabwe (i.e. the Gwayi, Manyame, Mzingwane, Runde, Sanyati, Mazowe and the Save catchment shown in Fig. 2) have been studied at varying degrees using various hydrological modelling techniques and tools. However, most of these studies have been done in the Zambezi basin catchments, i.e. the Mazowe and the Manyame catchments [144, 306, 314]. Catchments to the North-eastern and South-western part of the country, i.e. the Gwayi and the Save catchments have received very limited attention in terms hydrological modelling research over the years, while the Mzingwane catchment has had four prominent studies [196, 305, 315, 316] that directly applied modelling techniques over the past two decades showing a knowledge gap in this regard. Furthermore, in as far as the integration of land use and land cover change, and climate modelling in hydrological modelling studies is concerned, it was noted that very few studies, i.e. approximately 15% of hydrological studies done to date have attempted to advance knowledge in this direction. In other words, utilisation of advanced, coupled distributed physically based hydrological modelling techniques to expand the scope of understanding of the climate-land use-hydrology nexus in Zimbabwe has been very limited thus showing a huge knowledge gap in this regard. However, other non-modelling hydrological studies have been done in almost all catchments in the country covering various themes such as reservoir capacity and sedimentation rate estimation [279, 317], groundwater yield estimation [254], water quality assessment [318], in-field and rainwater harvesting [319, 320] and general catchment characterisation—water balance relationships [305, 315].

Though there has been tremendous advances in integration/streamlining of GIS and RS in hydrological modelling research globally over the past decades as earlier discussed [191, 298, 302], in Zimbabwe however, very few studies, e.g. [144, 145, 240, 254, 279, 306, 321] have applied these tools and techniques showing a need to expand knowledge in this area leveraging these techniques. This could be attributed to limited expertise and GIS and RS infrastructure/equipment to fully streamline the use of the techniques in hydrological modelling studies. This could also be exacerbated by the earlier highlighted challenges of limited accessibility and availability of good quality *in situ* climatic /meteorological data such as rainfall and temperature measurements in the country.

## Conclusion

### Climate change/variability studies

Despite the developments in climate and hydrological research, and the already confirmed climate impacts on human livelihoods, economies and general well-being and water resources in Zimbabwe, the scope of understanding of the climate-land use-hydrology interlink is still limited/poor. It has gaps as revealed in our study. Similarly, climatic conditions studies in Zimbabwe covered in this review present varying and in some instances contradictory conclusions though most agree that the climate has been changing or varying considerably in space and time with a temperature rise of less about 0.1 °C and an approximately 10% decrease per decade for rainfall over the 1900 to 1993 period. Follow up studies in this regard basically indicate the same temperature and rainfall trends though magnitudes of change have been varying and, in some instances, contradictory owing to the different methodologies used in these studies. It was noted that the use of different methodologies in the analysis of data in these studies further compounds the problem of comparability of findings. For example, some studies used simple parametric inferential statistics to test for significance of climatic trends while others used non-parametric techniques on the same. This basically shows the need for care in interpreting and/or comparing study findings in this regard. Furthermore, new and more robust climate trend analysis techniques have been developed over the years which can be utilised to re-interrogate the available climate datasets with more scientific rigor to close knowledge gaps related to biases and inaccuracies of some of the past studies covered in this review.

We can conclude that climate change and variability impact studies and climate vulnerability, adaptation and mitigation studies are the two-predominant categories of climate studies in Zimbabwe while climate modelling and governance study themes were the least covered. For climate impact studies, there has been greater bias towards agricultural and ecological impact themes with very limited coverage of energy and socio-economic climate impacts. Other themes that emerged included climate impacts on health and hydrological systems. Findings in this regard converged on this general conclusion asserted by the IPCC that Zimbabwe is a highly climate vulnerable country with limited resilience and poor adaptation policies and strategies in place to avert the inherent impacts of climate change and variability, notwithstanding the availability of relevant legislation and institutional framework, policy and strategies (e.g. the NCP, NAP, ZINGSA, ZCHPC and the NCCRS). Furthermore, considering that the global and regional climate forecasts indicate worsening of conditions, it is thus very important that climate science in Zimbabwe is updated to generate new and contextual knowledge leveraging on cutting edge recently developed tools and techniques rather than rely on outdated conclusions from past studies to inform climate policy formulation and strategy development for the country.

Furthermore, in this review, it emerged that climate modelling research is still a largely grey area in Zimbabwe. Most past studies have used GCMs and only a few have used RCMs with limited to no bias corrections and due consideration of the region-specific nature of most of these models. The implication of this are potential biases and errors and thus limited local applicability of some of their findings considering also recent developments and cautions in application of these tools at a local scale. This, thus, necessitates further expansion of knowledge on the same by leveraging on the potential presented by new and advanced Southern Africa regions-specific GCMs and RCMs such as the CCAM which have the ability to generate accurate climate perturbations at regional and local scale through advanced downscaling and bias correction techniques. New studies could expand knowledge by modelling impact scenarios within agriculture, biodiversity and hydrology such as surface run-off which influences overall water availability and thus security in Zimbabwe. Advancing knowledge in this regard will be vital especially for identifying, for example, the hydrologic consequences of changes in important climatic variables such as temperature, precipitation, and other landscape variables such as land use-land cover. This could contribute to holistic policy development and effective planning of current and future water management and security interventions. Furthermore, highly prohibitive costs of in situ current and historic climate and hydrological data imposed by governmental agencies such as the Zimbabwe National Water Authority (ZINWA) and the Meteorological Services Department are noted to be one of the potential serious bottlenecks impeding climate science research in the country. It is therefore important for the Government of Zimbabwe to address this by coming up with more pragmatic data-access policies that will make climate datasets more easily available and accessible to the Zimbabwean scientific community so as to encourage more research. This will allow for unhindered fast progress or advancement of climate science research in Zimbabwe, exploiting also the available national supercomputing capabilities at the ZCHPC.

### Hydrological modelling studies

Hydrological modelling is a relatively grey area of research in Zimbabwe with very few studies reviewed herein covering this research domain. Of the seven water catchments in Zimbabwe, the Manyame and the Mazowe catchments have received most attention as frontiers of hydrological modelling research in Zimbabwe whilst the Gwayi, the Runde, the Save and the Sanyati catchments have had least coverage. While some hydrological modelling research has been done on the Mzingwane catchment, the scientific knowledge is outdated, i.e. has been outpaced by advances in techniques and tools developed and used in this domain over the past two decades globally. With such knowledge gaps, *vis-à-vis* the already acknowledged highly vulnerable climate of Zimbabwe and the predicted worsening future climatic conditions in the country, it thus becomes very critical that deliberate efforts cascading from policy level, prioritise climate-hydrology modelling research in Zimbabwe. This is because all these aspects speak to present and future sustainable development in terms of water security and livelihoods.

Regarding the types of models, there is generally a need to test or apply new/advanced coupled hydrological models to better understand interlinkages between climate-hydrology and land use in Zimbabwe to update existing knowledge to be abreast with global and regional developments within this domain. In order to achieve this, approaches encompassing coupling of distributed hydrological models and properly downscaled GCM/RCM simulations as advocated for by various researchers should be considered. Such approaches could enhance understanding of local feedback mechanisms and interrelations between key natural-human systems influencing community livelihoods which is a specialised area of research within this domain. Furthermore, we note a relatively new and important frontier of climate-hydrology research, i.e. integration of indigenous knowledge systems (IKS) in the context of climate adaptation and mitigation in Zimbabwe which has to be encouraged and streamlined within this domain.

Overall, we conclude that climate science and hydrological modelling research in Zimbabwe is lagging behind vis-à-vis global and regional developments within these domains and thus the need to adopt a more systematic and holistic approach exploiting among other tools and techniques, coupled systems-based approaches (integrating climate-land use-hydrological modelling and GIS/RS) for better understanding of past, present and future climatic conditions and their hydrological impacts. This should be done without negating the need of developing new and/or fine-tuning the existing climate related and other relevant policy, legislative and institutional frameworks in Zimbabwe.
